# Effectiveness of a Theory-Informed Documentary to Reduce Consumption of Meat and Animal Products: Three Randomized Controlled Experiments

**DOI:** 10.3390/nu13124555

**Published:** 2021-12-20

**Authors:** Maya B. Mathur, Jacob R. Peacock, Thomas N. Robinson, Christopher D. Gardner

**Affiliations:** 1 Quantitative Sciences Unit and Department of Pediatrics, Stanford University, Stanford, CA 94305, USA; 2 The Humane League Labs, Rockville, MD 20849, USA; jacob@rethinkpriorities.org; 3 Stanford Solutions Science Lab, Department of Pediatrics, Stanford University, Stanford, CA 94305, USA; tom.robinson@stanford.edu; 4 Stanford Prevention Research Center, Stanford University, Stanford, CA 94305, USA; cgardner@stanford.edu

**Keywords:** meat consumption, dietary change, sustainability, education, behavior interventions

## Abstract

Several societal issues could be mitigated by reducing global consumption of meat and animal products (MAP). In three randomized, controlled experiments ( n=217 to 574), we evaluated the effects of a documentary that presents health, environmental, and animal welfare motivations for reducing MAP consumption. Study 1 assessed the documentary’s effectiveness at reducing reported MAP consumption after 12 days. This study used methodological innovations to minimize social desirability bias, a widespread limitation of past research. Study 2 investigated discrepancies between the results of Study 1 and those of previous studies by further examining the role of social desirability bias. Study 3 assessed the documentary’s effectiveness in a new population anticipated to be more responsive and upon enhancing the intervention content. We found that the documentary did not decrease reported MAP consumption when potential social desirability bias was minimized (Studies 1 and 3). The documentary also did not affect consumption among participants whose demographics suggested they might be more receptive (Study 3). However, the documentary did substantially increase *intentions* to reduce consumption, consistent with past studies (Studies 2 and 3). Overall, we conclude that some past studies of similar interventions may have overestimated effects due to methodological biases. Novel intervention strategies to reduce MAP consumption may be needed.

## 1. Introduction

Several exigent societal issues could be mitigated by reducing global consumption of meat and animal products (MAP) and encouraging predominantly plant-based diets in their place. Authoritative enjoinments for such a dietary shift have highlighted its potential to improve public health [1,2,3,4,5,6], reduce risks of zoonotic pandemics and antibiotic resistance [7], curb environmental degradation and climate change [3,4,5,6,8], and limit the preventable suffering and slaughter of approximately 500 to 12,000 animals over the lifetime of each human consuming a diet typical of their country [6,9]. Nevertheless, MAP consumption in Western countries far exceeds nutritional recommendations [3,6] and, worldwide, continues to rise substantially [4]. Whereas existing research attention has often focused specifically on reducing consumption of red and processed meat [10], in this paper, we focus more broadly on shifting consumption of all meats (i.e., edible animal flesh) and animal products (i.e., eggs and dairy) to plant-based diets. In contrast, shifting consumption from red and processed meat to poultry, fish, dairy, and eggs would be beneficial for individual health, but probably less so than making comparable shifts to healthy plant-based foods, such as vegetables, fruits, whole grains, and legumes, that are severely lacking in the standard Western diet [3,11,12]. Furthermore, producing poultry, fish, dairy, and eggs causes considerable environmental and ecological damage [13,14,15] and has severe animal welfare impacts [6].

Developing simple interventions to encourage dietary shifts from MAP to healthy plant-based foods could therefore carry widespread societal benefits. Educational interventions that make appeals to individual health [10,16], the environment [10,16], or animal welfare [16,17] may be effective. More subtle “nudge” interventions that may operate outside participants’ conscious awareness, for example by repositioning meat dishes to be less prominent in cafeterias, may also be effective [16,18]. Although these types of interventions are promising, many existing studies have methodological limitations [17]. These include the potential for social desirability bias that could artificially inflate apparent intervention effects [19], measurement of outcomes only in terms of participants’ attitudes or intended behavior rather than actual MAP consumption, and small sample sizes. As a result, we are aware of very few specific interventions that are adequately well-evidenced to strongly support their widespread dissemination at this point.

We conducted a series of parallel-group, randomized controlled experiments designed to help resolve these methodological challenges of previous studies. Namely, our studies took stringent precautions against social desirability bias, used longitudinal designs, and measured food consumption outcomes using food frequency questionnaires. The intervention was a 20-min documentary that encourages dietary shifts from all meats and animal products to plant-based diets. We selected this documentary because its content reflects certain best practices for designing effective interventions in general, and its content also harnesses the specific psychology of MAP consumption [17]. In general, **providing educational information** can influence beliefs and intentions that may subsequently shape behavior [20]. The public appears to be poorly informed about the aforementioned consequences of global MAP consumption; in fact, many individuals appear to deliberately avoid such information [21]. Thus, providing information that helps remedy this knowledge gap may be effective. Additionally, portraying the desired behavior as aligning **with social norms** (what others believe one should do, or what others actually do) can effectively shift behaviors, including food choices [22,23]. Providing concrete suggestions for how to change one’s behavior (e.g., recipes) may help individuals to form concrete **implementation intentions** for what they plan to do when faced with food choices [24]. According to the Theory of Planned Behavior, providing such suggestions may increase individuals’ perceived ability to control their future behavior and their intentions to do so [20]. Indeed, previous interventions to reduce consumption of meat and/or animal products that invoked these components have obtained preliminarily promising results. Such interventions have included, for example, providing leaflets, news articles, and videos [17,25,26,27].

In addition to leveraging these general components of effective behavioral interventions, the documentary we studied was designed to also harness the unique social, moral, and affective psychology underlying MAP consumption [28,29]. For example, although ethical concern about factory farming conditions is now a majority stance in several developed countries [30], MAP consumption remains nearly universal. This discrepancy between people’s ethical views and their actual behavior, termed the “meat paradox” [31], can induce cognitive dissonance. Previous interventions have successfully invoked this dissonance by using **meat-animal reminders**, which are simple visual or verbal reminders of the connection between MAP and animals (e.g., photographs of meat dishes presented next to photographs of the animals from which they came) [32,33,34,35,36,37]. Last, **physical disgust and moral disgust** are closely intertwined and powerfully shape food choices [38,39]. Experiencing physical disgust can amplify negative moral judgments, and conversely, experiencing moral disgust can induce physical disgust [40]. Previous interventions to reduce consumption of meat and/or animal products have often invoked disgust by describing, for example, “crowded conditions [and] pens covered in excrement and germs” [41]. We also selected this documentary because it has been disseminated in practice via social media advertising by a nonprofit, The Humane League. For example, in 2019, the nonprofit’s advertising generated 13 million visits to websites deploying documentary-driven interventions, including this documentary, resulting in 8 million minutes of viewing.

In Study 1, we aimed to assess the documentary’s effectiveness using a study design that improved upon certain methodological limitations of previous work, described above. In Study 2, we aimed to adjudicate discrepancies between the results of Study 1 and those of previous studies by further examining the role of social desirability bias. In Study 3, we aimed to assess the documentary’s effectiveness in a different population and upon adding new components to the intervention, which were designed to increase participant engagement. To this end, in Studies 1 and 3, our primary outcome was participants’ total MAP consumption over the past week (henceforth “consumption”), reported approximately 2 weeks after random assignment and exposure to the documentary. In both studies, we secondarily assessed consumption of specific categories of MAP as well as consumption of healthy plant-based foods. We also assessed the extent to which the intervention’s effects might differ by participants’ demographic characteristics; such findings could be used to cost-effectively target dissemination. For example, previous work has suggested that sex, education, and political liberalism could moderate the effectiveness of interventions to reduce consumption of meat and/or animal products [42,43,44,45]. Studies 1 and 3 took stringent precautions against social desirability bias. In Study 2, to further examine the potential for social desirability bias, our primary outcome was participants’ immediate intentions to increase, decrease, or not change their consumption, similar to many existing studies in the literature.

## 2. Study 1

### 2.1. Methods

For all 3 studies, we preregistered in detail all methods and statistical analyses, and the datasets and materials are publicly available (see “Data Availability Statement”). All studies were approved by the Stanford University IRB (protocol #57476). Statistical methods are detailed further in the Supplement. We conducted all statistical analyses in R [46], version 4.0.2.

#### 2.1.1. Study Design and Participants

We conducted a 2-arm, parallel-group, 12-day randomized controlled experiment comparing the documentary to an unrelated video, whose contents are detailed below. We conducted all studies online by embedding the videos in a questionnaire that we created in Qualtrics [47]. We used the online platform Prolific Academic to recruit United States-based participants who were at least 18 years old, without further demographic restrictions [48]. Prolific is a data-collection platform in which users can complete paid online research studies; the platform functions similarly to Amazon Mechanical Turk, but with apparently higher data quality [49]. Participants recruited through Prolific and similar platforms may be more demographically diverse than traditional undergraduate samples but are typically not a representative sample of the United States [49].

To maximize external generalizability and minimize social desirability bias, we used vague recruitment text that did not refer to MAP consumption or to motivations for reducing consumption [50]. Based on an *a priori* sample size determination (Supplement), we recruited 650 participants at the baseline wave of data collection ( $T_{0}$ ). To minimize social desirability bias by blinding participants to the study’s purpose, we used new recruitment text that described the follow-up wave ( $T_{1}$ ) as if it were a standalone study and did not reveal its connection to the $T_{0}$ wave. We refer to this approach as “**naïve re-recruitment**”. At $T_{1}$ , participants self-reported their consumption frequencies and typical serving sizes of 6 individual categories of meats (chicken, turkey, fish, pork, beef, other meat), 2 categories of animal products (dairy, eggs), 5 categories of healthy plant-based foods (leafy green vegetables, other vegetables, fruits, whole grains, legumes), and 2 decoy foods that were not analyzed (refined grains and sweetened beverages). We included the decoy foods to further conceal the purpose of the study. Participants also completed items that assessed their awareness of the purpose of the study and exploratory attitude measures, detailed below. All questionnaire items appear in the Supplementary Materials.

#### 2.1.2. Intervention Documentary and Control Video

The intervention was a 20-min documentary, *Good For Us*, produced by The Humane League and designed with close attention to psychological theory [51]. The documentary encourages plant-based diets that reduce consumption of all meats and animal products, thus differing from many existing interventions that focus on reducing consumption of red and processed meat in favor of not only plant-based foods but also poultry, fish, dairy, and eggs [10]. The documentary’s broader focus aligns with the holistic societal concerns that motivated this research, as described in the Introduction.

The documentary was designed to shift explicit attitudes and intended behaviors [20] using educational appeals to individual health, to the environment and climate change, and to animal welfare. Through its multiple narrators, the documentary makes recommendations to eat, for example, “plant-based” and “vegetarian, and better yet, vegan” diets. In addition to these direct educational appeals, the documentary uses indirect means to shift dietary behavior, as discussed in the Introduction. First, the documentary invokes physical disgust by showing graphic footage of factory farms and slaughterhouses (e.g., of laying hens in filthy battery cages). Second, the documentary uses both verbal and visual meat-animal reminders (e.g., by cutting directly from footage of broiler chickens in a factory farm to footage of packaged chicken in a supermarket). Third, the documentary invokes social norms regarding increased demand for plant-based meals. For example, a narrator states: “Restaurants are catering to the millenial population that is really demanding...a vegan diet, a vegetarian diet, a flexitarian diet.” As described in the Introduction, psychological theory and empirical findings suggest that these elements may be potent means of reducing MAP consumption [17]. We added to the end of the documentary a brief screen stating, “For practical tips on shifting to plant-based eating, see: www.eatingveg.org/how and www.eatingveg.org/what.” These websites provide, for example, recipes and tips for handling social pressure from family and friends. (These websites have since been moved and modified. Examples of content similar to what participants would have seen at those URLs are publicly available at https://osf.io/xrckh/). Providing such practical tips may help individuals to form concrete implementation intentions, as discussed in the Introduction.

For the control group, we opted to present a control video rather than no video in order to hold constant the duration of study between intervention and control participants, thus reducing the possibility of differential dropout between these groups. The control video was a 20-min TED talk by Brené Brown entitled “Listening to shame”, which was a generic motivational speech that encouraged listeners to embrace the experience of feeling vulnerable. The content was unrelated to food choices and to any of the aforementioned reasons for reducing MAP consumption. We chose this video because its length matched that of the documentary and because pilot studies indicated that, like the documentary, participants found it engaging.

#### 2.1.3. Outcomes

Participants reported their past week’s consumption of each of the 6 categories of meats, 2 categories of animal products, 5 categories of healthy plant foods, and 2 decoy foods using a modified version of the National Cancer Institute’s Dietary Health Questionnaire III (DHQ III). This scale was designed to reduce measurement error, and validation studies comparing versions of the DHQ to 24-h recalls have estimated that these measures had deattenuated correlations of approximately 0.50 for meats and eggs, and approximately 0.75 for dairy [52,53,54]. We modified the 1-month DHQ-III that includes portion sizes, as follows: (1) we adapted the frequency options to correspond to our briefer follow-up time frame; (2) to reduce survey fatigue, we collapsed individual foods into fewer categories, informed by those used in [26]; and (3) we omitted a large number of foods not relevant to the present research.

For each food category, participants answered the question, “Over the past week, how often did you eat [food category]?” using 6 ordinal responses ranging from “never” to “2 or more times per day”. Participants also reported the weights of foods consumed by answering the question “Each time you ate [food category], how much did you usually eat?” using 3 ordinal responses (“less than 2 ounces or less than 1/2 cup”, “2 to 5 ounces or 1/2 to 1 cup”, or “more than 5 ounces or more than 1 cup”). We estimated the total amount of each food category consumed by each participant by multiplying consumption frequencies by weights. Participants’ total weight of MAP consumed over the past week was the primary food outcome, and their consumption of the individual foods were secondary outcomes.

We also collected exploratory measures of participants’ attitudes and values regarding health, the environment, and animal welfare [55]. We also probed in more detail participants’ attitudes regarding animal welfare because this type of educational appeal has appeared only relatively recently in dietary interventions [17]. These exploratory measures are detailed in the Supplement.

#### 2.1.4. Other Measures

At $T_{0}$ , participants provided their sex, age, education level, race/ethnicity, current state and county of residence, and political party affiliation. We calculated an index of “county liberalism”, representing the proportion of Democratic votes in the participant’s county (Supplement). These measures were collected as possible moderators of intervention effects and for use in multiple imputation (Section 2.1.5). We did not measure consumption at baseline because doing so could have increased participants’ awareness of the purpose of the study. Measuring baseline consumption was not necessary because randomization ensures balance between groups, on average, on this variable and other potential confounders.

As an attention check, we asked participants, “Which of the following points did the video make? Please select all that apply.” Participants could select any number of statements from among 5. Exactly one statement was correct for the intervention group (“The ways we raise animals for human consumption causes the animals to suffer”) but was incorrect for the control group. The remaining 4 statements were plausible, but were incorrect for both groups (e.g., “Most Americans get less than the recommended amount of exercise”). Thus, if participants were perfectly attentive, all intervention participants would choose exactly one answer, while all control participants would not indicate any of the answers.

At $T_{1}$ , for use in sensitivity analyses (Section 2.1.5), we assessed participants’ potential awareness of the study’s purpose using 2 multiple-choice items that mimicked a funnel debriefing (Supplement). We coded participants as “potentially aware” if their responses indicated that they correctly believed that the researchers intended to decrease MAP consumption. Additionally, because we conducted this study during the SARS-CoV-2 pandemic, we asked participants to what extent the pandemic was affecting their ability to choose what they eat; this item was simply descriptive.

#### 2.1.5. Statistical Analyses

##### Analysis of Primary and Secondary Outcomes

We estimated the difference in mean total consumption between the intervention and control group and conducted a 2-sample Welch’s *t*-test, which accommodates the heteroskedasticity and skewed errors that are typical of food outcome measures [56,57]. We repeated this analysis for the secondary consumption outcomes and exploratory attitude outcomes. We calculated *p*-values for all secondary outcomes both with and without Bonferroni correction, counting one test per secondary outcome (corrected α=0.05/17 tests = 0.0029) [58]. We tested the global null hypothesis that the intervention affected none of the secondary outcomes by calculating harmonic mean *p*-values [59] for all secondary outcomes considered together, for all secondary food outcomes considered together, and for all exploratory attitude outcomes considered together. (Harmonic mean *p*-values aggregate potentially non-independent *p*-values and represent an outcome-wide metric of the intervention’s effect on the secondary outcomes [60].) We also calculated the number of secondary outcomes with a Bonferroni-corrected p<0.05 , which can be interpreted with 95% confidence as the number of secondary outcomes on which the intervention has a nonzero effect [61]. Throughout these analyses, we multiply imputed missing data that arose from attrition at $T_{1}$ [62,63].

##### Analysis of Moderators

We examined 2-way interactions of the intervention with baseline participant characteristics: being female, being ≤25 years old, having at least graduated from a 2-year college, being a Democrat (vs. being a Republican), being an Independent or reporting no party affiliation (vs. being a Republican), being Caucasian, and county liberalism (rescaled to represent a 10-percentage point higher share of Democratic votes in the participant’s county). We collapsed non-Caucasian race categories because those categories contained few individuals (Table 1). We included all of these candidate moderators simultaneously in a generalized least-squares model with heteroskedasticity-consistent robust standard errors [56]. We again reported inference both with and without Bonferroni correction, counting one test per moderator regression coefficient.

##### Sensitivity Analyses

We conducted 3 sensitivity analyses for the primary results. First, we conducted a complete-case analysis as a counterpart to the primary multiple imputation analyses. Second, anticipating that there may be more nondifferential measurement error (i.e., random noise) in participants’ reporting of serving size volumes than in their reporting of consumption frequencies (e.g., because participants may have difficulty estimating volumes of food), we repeated the primary analysis using frequencies alone as the outcome, rather than total amounts consumed. Third, we accounted for possible inattention to the intervention by treating the intervention assignment as an instrumental variable for passing the attention check [64]. This analysis makes an important statistical assumption of excludability, as we detail in the Supplement.

### 2.2. Results

#### 2.2.1. Participant Characteristics

We randomized 649 participants at $T_{0}$ (327 in the intervention group and 322 in the control group; Table 1). (Due to a technical glitch, one participant had a duplicated record in which they had completed the questionnaire twice. We excluded this participant’s second record without compromising intention-to-treat principles because the duplicate record did not represent a unique participant.) The sample was roughly balanced on sex, and compared to overall United States national demographics [65], was somewhat younger (median 31 years), more educated (with 58% having at least graduated from a 4-year college), and considerably more politically liberal (49% Democrats versus 24% Republicans). At $T_{1}$ , 574 participants completed data collection (88% retention); individual follow-up times between $T_{0}$ and $T_{1}$ had a mean and median of 12 days. Retention was nearly identical for the intervention and control groups (88% and 89% respectively). A plurality of participants (49%) indicated that the SARS-CoV-2 pandemic had not changed their ability to choose what they eat.

#### 2.2.2. Attention Check and Awareness of Study’s Purpose

Among intervention-group participants, 95% correctly indicated that the intervention had discussed farm animal welfare concerns while possibly also indicating other incorrect answers. In contrast, only 14% of participants in the control group chose this answer (i.e., they happened to guess correctly). As a more stringent consideration, 76% of participants in the intervention group chose the single correct answer and no others (i.e., they passed the attention check), compared to 9% of control participants who guessed correctly. Only 3% of intervention-group participants were potentially aware of the purpose of the study, which was similar to the proportion of participants in the control group who guessed correctly (1%).

#### 2.2.3. Effect of the Documentary on Outcomes

Table 2 shows the documentary’s estimated effects on all outcomes. The documentary did not reduce total 1-week consumption compared to the control video ( −0.33 oz/week; 95% CI: [ −6.12 , 5.46]; p=0.91 ; standardized mean difference [ SMD ] = −0.01 ; 95% CI: [ −0.17 , 0.15]). The documentary also did not meaningfully affect any of the secondary food outcomes or the exploratory attitude outcomes: most standardized mean differences were very close to zero and all were less than 0.20 in magnitude. None of the Bonferroni-corrected *p*-values for the secondary outcomes was less than 0.05. As outcome-wide measures of the intervention’s effect, the harmonic mean *p*-values were p=0.66 for all secondary outcomes considered together, p=0.90 for the secondary food outcomes, and p=0.23 for the exploratory attitude outcomes. Given these null results, we did not pursue certain cost-effectiveness analyses that we had planned.

#### 2.2.4. Moderators

Table 3 shows estimated differences in the documentary’s effectiveness for each candidate moderator, along with all main-effect estimates. The results did not support moderation by these variables, at least when considered individually. Demographic characteristics whose estimate direction was consistent with improved intervention effectiveness were, in descending order of estimate magnitude: living in a more politically liberal county (by 10 percentage points), being female, having completed at least 2-year college, being a Democrat (vs. a Republican), being Caucasian, being politically Independent/other, and being ≤25 years old. These findings regarding sex and education [44] and political liberalism [45] are directionally consistent with previous literature.

#### 2.2.5. Sensitivity Analyses

Conducting complete-case analyses on only the 574 participants who completed data collection at $T_{1}$ yielded similar results to using multiple imputation (Supplementary Table S1). The intervention also did not change participants’ reported total frequencies of consuming MAP ( −0.34 consumption instances per week; 95% CI: [ −1.81,1.14 ]; p=0.65 ; SMD=−0.04 ; 95% CI: [ −0.19,0.12 ]. The instrumental variables analysis suggested that the intervention might have been effective among those participants who passed the attention check (estimated intervention effect of −8.78 oz/week; 95% CI: [ −17.28,−0.27 ]; p=0.043 ; SMD=−0.24 ; 95% CI: [ −0.48,−0.01 ]. Because the primary analyses did not indicate an intervention effect, we did not pursue a planned sensitivity analysis regarding the severity of social desirability bias that would be required to explain away the effect.

### 2.3. Discussion

These null results stand in contrast to those of recent reviews and meta-analyses [10,16,17]. The discrepancy could reflect a genuine difference in effectiveness if, for example, the specific documentary we tested was less effective than existing interventions, or if the participants we recruited were less receptive to its effects. Alternatively, the discrepancy might be an artifact of differences in biases affecting our estimates versus those in existing studies. Most existing studies measured outcomes in terms of participants’ immediate, reported intentions to reduce consumption, but immediate intentions may not accurately predict actual consumption for at least 2 reasons [17]. First, participants may not follow through on their genuine intentions [66]. Second, when participants are aware of the study’s purpose, the intervention might bias some participants’ reported intentions (i.e., social desirability bias). We sought to reduce these biases by measuring outcomes after 12 days, by using naïve re-recruitment, and by including decoy food items. Our analyses suggested that these precautions did successfully blind participants to the study’s purpose. To specifically investigate the extent to which these biases might have contributed to the discrepancy in results, we conducted Study 2, which was designed to resemble the majority of existing studies.

## 3. Study 2

### 3.1. Methods

#### 3.1.1. Study Design and Participants

We recruited 300 participants on Prolific. The recruitment strategy and study design were identical to Study 1 except for 2 key differences. First, we collected outcome data immediately after showing participants the documentary or control video. Second, consistent with the immediate assessment of outcomes, participants reported their intentions using a single item similar to those used in existing studies [26,67,68,69]: “How is your consumption of meat and animal products likely to change over the next 7 days?” Participants answered using a 7-point Likert scale ranging from “strongly decrease” to “strongly increase”. We reasoned that if the documentary were to remain ineffective when using this outcome measure, then presumably the documentary was genuinely less effective in our Study 1 than similar interventions were in existing studies (e.g., due to characteristics of the intervention itself or differences in the samples studied). On the other hand, if the documentary were to appear more effective when using this outcome measure, this might instead suggest that immediate intentions are not accurate proxies for consumption in the context of intervention studies on reducing MAP consumption.

Following this new primary outcome item, participants answered all items from Study 1’s follow-up wave. Critically, the food consumption items still asked participants to report their consumption over the past week, even though Study 2 assessed outcomes immediately after exposure to the assigned video. We reasoned that any apparent intervention effects on these self-reported measures regarding behavior *before* random assignment and exposure to the documentary or control video would suggest social desirability bias. We term this “**spurious retrospective causation**”.

#### 3.1.2. Statistical Analyses

We repeated the analyses of Study 1, omitting sensitivity analyses. We analyzed the new intention outcome on 2 scales: treated as pseudo-continuous ( −3 = “strongly increase” to 3 = “strongly decrease”, with 0 representing no change) and also dichotomized to represent intending to reduce consumption versus intending to increase or not change consumption. The latter binary measure provides an effect size whose scale is directly comparable to the results of a previous meta-analysis [17]. For the continuous intention measure, we calculated a mean difference and estimated inference with a 2-sample Welch’s *t*-test; for the binary measure, we calculated a risk ratio and estimated Wald-type inference.

### 3.2. Results

We randomized 300 participants (148 to the documentary and 152 to the control video) and made no exclusions. The participants were demographically very similar to those in Study 1, as expected given the identical recruitment strategies (Supplementary Table S2). Participants in the control group on average reported that they did not intend to change their consumption after watching the control video ( 0.23 points on the Likert scale from −3 to 3), whereas participants in the intervention group on average reported that they intended to “somewhat decrease” consumption after watching the documentary (0.99 points). Thus, the documentary increased participants’ reported intentions to reduce MAP consumption by 0.76 points (95% CI: [0.49, 1.02]; p<0.0001 ; SMD=0.65 ; 95% CI: [0.41, 0.88]) compared to the control video. Similarly, 20% participants in the control group reported that they intended to reduce consumption, compared to 68% in the intervention group. Thus, the documentary increased the percentage of participants intending to reduce consumption by 3.42-fold (95% CI: [2.49, 4.92]; p<0.0001 ). As in Study 1, there was little indication of moderation, although Study 2 was not well-powered to detect such effects (Supplementary Table S3).

Regarding spurious retrospective causation, the documentary did not substantially affect participants’ reported total consumption over the week before random assignment ( −4.07 oz/week; 95% CI: [ −11.74,3.60 ]; p=0.30 ; SMD=−0.12 ; 95% CI: [ −0.35,0.11 ]), though the point estimate was in the direction consistent with social desirability bias. Estimates for individual foods appear in Supplementary Table S4.

### 3.3. Discussion

Whereas Study 1 suggested that the documentary had little, if any, effect on participants’ reported consumption at a 12-day follow-up, Study 2 suggested that the documentary substantially increased participants’ immediate intentions to reduce consumption. Given the nearly identical sampling frames, a likely explanation is that the documentary did affect participants’ stated intentions to reduce consumption, but these intentions had little influence on subsequent self-reported behavior. Alternatively, intentions assessed immediately after the intervention might be particularly susceptible to social desirability bias. In Study 3 below, we used a design similar to that of Study 1. We enhanced the intervention by adding additional questionnaire items to increase participants’ engagement with the documentary content. Additionally, rather than recruiting participants on Prolific, we recruited from a university registry of participants in nutrition research. We speculated that the registry participants might be more receptive to the intervention and attentive than Prolific users.

## 4. Study 3

### 4.1. Methods

#### 4.1.1. Study Design and Participants

The study design was similar to that of Study 1, with the following differences. First, we recruited participants from a Stanford University Prevention Research Center registry comprising previous participants of nutrition studies. These registry participants had either previously participated in nutrition studies or had inquired about participating in such research. We invited all 8346 members of the registry to participate in the $T_{0}$ questionnaire. At $T_{1}$ (14 days after $T_{0}$ ), we invited all participants who had completed $T_{0}$ to complete the $T_{1}$ questionnaire containing the outcome measures. As in Study 1, we again used naïve re-recruitment (i.e., describing the $T_{1}$ questionnaire as if it were an unrelated study) as a precaution against social desirability bias.

Second, we examined moderation by participant demographics in a confirmatory rather than exploratory manner. In Study 1, we had estimated that 2 demographic characteristics that were directionally consistent with increased intervention effects were having completed at least 2-year college and being a Democrat. In Study 2, we hypothesized that the intervention might be more effective for participants with these 2 characteristics (henceforth participants in the “target demographic”). To improve power, we stratified randomization on whether a participant was in the target demographic.

#### 4.1.2. Intervention

In this study, we also enhanced the intervention by introducing new questionnaire items designed to increase participant engagement with the documentary content (Supplement). We presented the documentary as 3 sequential segments corresponding to content about health, the environment, and animal welfare, and presented in the same order as in the original intervention. Each segment was followed by free-response questions about the content presented (e.g., “As discussed in the video, plant-based diets can have several health benefits. Can you name at least 2 of these benefits that you found compelling?”).

After the documentary, but not the control video, we asked participants whether they would like to pledge to reduce their consumption, pledge to eliminate their consumption, or to make no pledge regarding their consumption of each of the 7 categories of meats and animal products. These pledges conceptually resembled those of a previous study, in which willingness to take certain pledges mediated the intervention effect [70]. To additionally invoke social norms [22,23,71], this questionnaire item also stated that “Many of our previous Stanford research participants have pledged to eat and drink less meat and animal products after watching this documentary”, a statement that appeared to be true based on pilot studies.

For participants who were assigned to view the documentary and who had chosen to make at least one “reduce” or “eliminate” pledge (henceforth “pledge-making participants”), we then presented several items designed to improve general and specific goal-setting and self-monitoring, which can be effective components of lifestyle behavior-change interventions in general [72]. These items asked pledge-making participants to choose a specific date by which they intended to meet their pledge goals, asked them to select specific strategies they intended to use to fulfill their pledge (e.g., “I will choose a specific day of the week (e.g., Monday) when I will eat only plant-based meals”), and finally suggested concrete ways that participants could track their progress meeting their pledge goals (e.g., smartphone apps). One week after $T_{0}$ , we sent a customized email to pledge-making participants. Based on each participant’s previous questionnaire responses, this email reminded the participant of the specific food(s) they had chosen to reduce or eliminate from their diet, of the date on which they intended to meet their pledge goals, and of the strategies they intended to try. These emails were informed by literature on lifestyle behavior-change interventions, which suggests the importance of prompting participants to review their behavioral goals and to monitor their progress toward meeting these goals [72].

#### 4.1.3. Outcome Measures

The primary and secondary outcome measures were the same as in Study 1. That is, the primary outcome was self-reported MAP consumption, as assessed 14 days after random assignment ( $T_{1}$ ) on a food frequency questionnaire.

#### 4.1.4. Statistical Analyses

The analyses were as in Study 1, with the following changes. In all analyses, we statistically controlled for the variables used to stratify randomization to obtain correct statistical inference (except in models that contained only participants in the target demographic, described below) [73]. To do so, we used regression with heteroskedasticity-consistent robust standard errors. These regression models included covariates representing intervention assignment and being in the target demographic.

We also conducted 2 analyses to examine moderation by membership in the target demographic. For the first analysis, we revised the primary analysis model to include an interaction term of membership in the target demographic with intervention assignment. For the second analysis, we estimated the intervention effect within only participants in the target demographic.

### 4.2. Results

#### 4.2.1. Participant Characteristics

We randomized 665 participants (333 to the documentary and 332 to the control video) and made no exclusions. Table 4 shows their demographic characteristics. Compared to the participants of Studies 1 and 2, these participants were more frequently female (73%), older (median 59 years), more highly educated (with 83% having at least graduated from a 4-year college), and even more politically liberal (60% Democrats and 4% Republicans).

At $T_{1}$ , 217 participants completed data collection (33% retention). This was a lower retention rate than was achieved in Study 1, although retention in Study 3 remained comparable for the intervention and control groups (30% and 35% respectively). To explore whether retention was related to participant demographics, we regressed an indicator for retention at $T_{1}$ on all demographic characteristics collected at $T_{0}$ as well as the intervention group. This model did not strongly predict retention ( p=0.13 ), suggesting that retention did not differ substantially across participant demographics or intervention group.

#### 4.2.2. Attention Check and Awareness of Study’s Purpose

At the end of $T_{0}$ , almost all participants in the intervention group (98%) correctly indicated that the intervention had discussed farm animal welfare concerns while possibly also indicating other incorrect answers; no participants in the control group chose this answer. (For comparison, Study 1 found 95% and 14%, respectively.) More stringently, 92% of participants in the intervention group chose the single correct answer and no others (i.e., passed the attention check), compared to 0% of control participants. (Study 1 found 76% and 9%, respectively.) Regarding potential social desirability bias, 13% of participants in the intervention group were potentially aware of the purpose of the study, versus 5% of participants in the control group who guessed correctly.

#### 4.2.3. Effect of the Documentary on Outcomes

The enhanced intervention did not meaningfully reduce total 1-week consumption compared to the control video ( −2.46 oz/week; 95% CI: [ −8.78,3.85 ]; p=0.43 ; SMD=−0.09 ; 95% CI: [ −0.32,0.14 ]); however, the fairly wide confidence intervals indicated moderate uncertainty. The enhanced intervention also did not appear to affect any of the secondary food outcomes or the exploratory attitude outcomes (Table 5): most standardized mean differences were close to zero and all were less than 0.15. All secondary outcome *p*-values were 1 after Bonferroni correction. The harmonic mean *p*-values were p=0.95 for all secondary outcomes considered together, p=0.86 for the secondary food outcomes, and p=0.86 for the exploratory attitude outcomes.

#### 4.2.4. Effect of the Documentary among Participants with Target Demographics

The intervention was also not effective among the subset of participants in the target demographic ( −1.72 oz/week; 95% CI: [ −8.84,5.41 ]; p=0.63 ; SMD=−0.07 ; 95% CI: [ −0.34,0.21 ]. Corroborating this, the estimated interaction between membership in the target demographic and intervention assignment was small and in the unexpected direction (1.55 oz/week; 95% CI: [ −7.76,10.85 ]; p=0.74 ; SMD=0.06 ; 95% CI: [ −0.28,0.40 ]). However, the confidence intervals for this analysis were quite wide.

#### 4.2.5. Intervention Engagement Items

Of the 332 intervention-group participants, 36% pledged to eliminate consumption of at least one meat or animal product, 57% made at least one pledge to reduce consumption, and 60% made at least one pledge of either type. Table 6 disaggregates percentages by pledge type and food type.

#### 4.2.6. Sensitivity Analyses

Complete-case analyses of participants who completed data collection at $T_{1}$ ( n=217 ) yielded a somewhat stronger intervention effect than primary analyses (Supplementary Table S5). The instrumental variables analysis suggested that the intervention effect among attentive participants was approximately twice as strong as that seen in the primary analysis ( −5.33 oz/week; 95% CI: [ −14.44,3.78 ]; p=0.24 ; SMD=−0.20 ; 95% CI: [ −0.53,0.14 ]), but the confidence interval was very wide.

### 4.3. Discussion

This study corroborated the results of Study 1, this time with an enhanced intervention and in a new population of participants, who were more attentive and likely more strongly motivated by health considerations. For example, among control-group participants, average ratings for the importance of health (which was one of the exploratory attitude outcomes) were 4.23 in Study 3 versus 3.88 in Study 1. Based on our moderator analysis from Study 1, these participants’ demographics also suggested that they might be more receptive to the intervention. However, even in these favorable conditions, we did not observe an improvement in the enhanced intervention’s effectiveness. Additionally, there was little evidence that the intervention was effective even among participants in the target demographic. The modest retention rate in this study tempers conclusions, although it is somewhat reassuring that intervention assignment and demographics did not strongly predict retention. Additionally, participants at $T_{1}$ were still largely unaware of the purpose of the study. We used statistical methods that can reduce some, but not all, forms of bias due to missing data; nevertheless, missing data still reduces statistical power.

## 5. General Discussion

In a series of 3 randomized, controlled experiments, we investigated the effectiveness of a documentary that was professionally designed to reduce MAP consumption by harnessing many of the best practices in designing interventions to change behavior. The documentary’s content was designed to leverage general components of effective behavioral interventions (e.g., by providing educational information, invoking social norms, and providing implementation suggestions) and of the specific psychology governing MAP consumption (e.g., by invoking physical and moral disgust and using meat-animal reminders).

In a study designed to minimize social desirability bias (Study 1), we found that the documentary did not affect participants’ reported consumption 12 days after random assignment ( −0.33 oz/week; 95% CI: [ −6.12,5.46 ]). However, in a second study with less protection against social desirability bias (Study 2), the documentary did substantially increase the percentage of participants who immediately intended to reduce consumption from 20% to 68%, a 3.42-fold change (95% CI: [2.49, 4.92]), which was in fact an even larger effect size than was seen in existing, comparably designed, studies and meta-analyses [17]. Finally, we changed the study sample from members of a commercial study recruitment platform to participants who had previously volunteered to be contacted about nutrition studies, and we enhanced the intervention with evidence-based engagement items and opportunities to form concrete goals (Study 3). These changes did not markedly improve the intervention’s effects on subsequently reported MAP consumption ( −2.46 oz/week; 95% CI: [ −8.78,3.85 ]), even though a majority of participants who viewed the documentary (60%) had pledged to reduce or eliminate their consumption of at least one meat or animal product. Additionally, the documentary was not substantially more effective among participants who were expected to be more receptive to its content: among participants who had expressed interest in participating in nutrition studies (i.e., all participants in Study 3) or among the demographic subset of these participants who were Democrats and had at least graduated 2-year college. The documentary also did not meaningfully affect participants’ responses to secondary consumption outcomes or the exploratory attitude outcomes.

### 5.1. Strengths and Limitations

This research has a number of methodological and conceptual strengths. To help mitigate widespread limitations of previous research regarding the potential for social desirability bias [17], we introduced methodological innovations to help prevent and benchmark social desirability bias in studies of reported food outcomes. First, in the 2 longitudinal studies (Studies 1 and 3), we used naïve re-recruitment to conceal the purpose of the study. We also included decoy foods (e.g., sweetened beverages) in the outcome measurements. Awareness probes in these studies indicated that, with these methods, participants indeed remained largely unaware of the purpose of the experiment during follow-up data collection. In Study 2, as a benchmark of the severity of social desirability bias, we investigated “retrospective causation” by asking participants to report on their past-week consumption, mere minutes after they had viewed either the documentary or the control video. Any apparent intervention effects on reported consumption over the week *prior* to random assignment would seem to necessarily reflect social desirability bias. We estimated that the documentary affected these retrospective reports of total consumption by −4.07 oz/week (95% CI: [ −11.74,3.60 ]). Although the confidence interval is wide, this spurious point estimate is in the vicinity of typical intervention effects seen in studies of interventions to reduce meat consumption, most of which had similar designs that are susceptible to social desirability bias [17].

As an additional methodological strength, in Studies 1 and 3, we assessed reported consumption rather than only intentions, and we assessed consumption in terms of the numerical weight of food that participants reported consuming of specific categories of meats and animal products (e.g., chicken, beef). Such measures would allow intervention effects to be translated into direct measures of cost-effectiveness and societal impact, such as the estimated reduction in human all-cause mortality events, in the number of animals raised for consumption, and in greenhouse gas emissions [17]. Last, in Study 3, we enhanced the intervention with engagement items that were based on strong evidence from the broader literature on lifestyle behavior-change interventions, including tailored content via the interim emails reminding participants of their stated pledges [72]. These enhancements have rarely been studied in the context of reducing MAP consumption [17].

This research also has limitations. Although our findings seem to rule out the possibility that this intervention has large effects, smaller effects may nevertheless be consequential if a large number of individuals were exposed to the intervention, or if effects accumulated over repeated exposures [74]. Indeed, we intentionally selected an intervention that has already been widely disseminated at relatively low cost. However, it is difficult to precisely establish small intervention effects without very large sample sizes and high retention. Although Study 1 achieved high retention, Study 3 did not, which compromises statistical precision and increases susceptibility to missing data bias. Additionally, reported dietary data can be subject to substantial measurement error even when collected via measures such as 24-h recalls or food frequency questionnaires [75,76]; such measurement error can further obscure small intervention effects. Although Studies 1 and 2 used somewhat longer durations of follow-up (approximately 2 weeks) than most existing studies [17], even longer durations would help identify potential delayed adoption and would characterize how well effects are sustained over time. The documentary we studied was previously disseminated by a nonprofit, so it is possible that some individuals may already have viewed it prior to participating in our studies, although no participants mentioned having seen the documentary when they provided free-text feedback on the study. Finally, it is possible that the control video could have elicited effects of its own, although this seems unlikely given its irrelevant content.

### 5.2. Future Directions

The majority of previous studies of similar interventions have assessed outcomes in terms of participants’ reported intentions to change their consumption, using designs similar to our Study 2 [17]. Indeed, in this context, the documentary we studied appeared to be remarkably effective, with an effect size stronger than those of any of the 100 studies included in our recent meta-analysis [17]. That is, in Study 2, we estimated a 3.42-fold reduction in the percentage of participants who intended to reduce consumption. Critically, though, the documentary had little effect on reported consumption after approximately 2 weeks. This discrepancy between intentions and reported consumption reiterates that reported intentions may be a poor proxy for reported actual consumption, and further underscores the urgency of designing studies that minimize social desirability bias. Future longitudinal studies could consider adopting the same methodological innovations, detailed above, that we used to successfully reduce participant awareness.

As noted above, precisely estimating potentially small, but consequential, intervention effects requires large sample sizes as well as stringent precautions against biases and sources of random measurement error. Future studies of similar interventions might consider using designs other than traditional individual randomization. For example, interventions could be deployed at locations of food purchases (e.g., by posting educational flyers in a grocery store). This could facilitate measuring outcomes in terms of actual food purchases at that location and could also help generate social norms within local groups. Such designs can enable large sample sizes while reducing the potential for social desirability bias and measurement error [77].

In conclusion, these findings suggest that some past studies of similar interventions may have overestimated effects due to methodological biases. The methodological innovations we introduced could help future studies mitigate these biases. Novel intervention strategies may be needed to effectively shift dietary consumption away from MAP.

## Figures and Tables

**Table 1:** For Study 1, demographic characteristics of the 649 participants at baseline. Continuous variables are reported as medians with 25th and 75th percentiles. Binary variables are reported as counts and percentages. Subjects could indicate multiple races. “County liberalism”: in the subject’s county, the proportion of votes from the 2000–2016 United States presidential elections that went to the Democratic candidate.

Characteristic	Intervention (*n* = 327)	Control (*n* = 322)
**Sex**
Male	164 (50%)	178 (55%)
Female	158 (48%)	140 (43%)
Other	5 (2%)	4 (1%)
**Age (years)**	30 (24, 41)	32 (23, 41)
**Education**
Did not graduate high school	0 (0%)	2 (1%)
Graduated high school	102 (31%)	103 (32%)
Graduated 2-year college	36 (11%)	28 (9%)
Graduated 4-year college	116 (35%)	119 (37%)
Completed post-graduate degree	73 (22%)	70 (22%)
**Political party**
Democrat	149 (46%)	171 (53%)
Republican	82 (25%)	76 (24%)
Independent	78 (24%)	61 (19%)
Other/I don’t know	18 (6%)	14 (4%)
**County liberalism**	0.57 (0.45, 0.70)	0.55 (0.43, 0.70)
**Race**
Caucasian	242 (74%)	229 (71%)
Black/African American	32 (10%)	25 (8%)
Hispanic	26 (8%)	30 (9%)
East Asian	24 (7%)	30 (9%)
Southeast Asian	9 (3%)	13 (4%)
South Asian	12 (4%)	11 (3%)
Native American	8 (2%)	9 (3%)
Middle Eastern	2 (1%)	6 (2%)
Pacific Islander	3 (1%)	3 (1%)

**Table 2:** In Study 1, estimated intervention effects for the primary outcome, secondary food outcomes, and exploratory attitude outcomes. Raw mean differences represent ounces consumed over the past week for the primary outcome and secondary food outcomes; they represent units on a 7-point Likert scale for the perceived importance items; and they are omitted for the three composite scales, which were already standardized. Brackets are 95% confidence intervals without correcting for multiple testing.

Outcome	Raw Mean Difference	Standardized Mean Difference	*p*-Value	Bonferroni *p*-Value
**Primary outcome**
Total meat and animal products	−0.33 (−6.12, 5.46)	−0.01 (−0.17, 0.15)	0.91
**Secondary food outcomes**
Meat	−1.14 (−5.25, 2.97)	−0.04 (−0.2, 0.11)	0.59	1
Non-meat animal products	0.82 (−2.43, 4.07)	0.04 (−0.13, 0.21)	0.62	1
Chicken	−0.01 (−1.98, 1.97)	0.00 (−0.16, 0.16)	1	1
Turkey	−0.5 (−1.42, 0.41)	−0.09 (−0.26, 0.08)	0.28	1
Fish	0.00 (−1.03, 1.04)	0.00 (−0.16, 0.16)	1	1
Pork	−0.1 (−0.98, 0.78)	−0.02 (−0.18, 0.14)	0.82	1
Beef	−0.39 (−1.62, 0.84)	−0.05 (−0.21, 0.11)	0.53	1
Other meat	−0.16 (−0.95, 0.64)	−0.03 (−0.19, 0.13)	0.7	1
Dairy	1.09 (−1.72, 3.9)	0.07 (−0.11, 0.24)	0.45	1
Eggs	−0.27 (−1.63, 1.09)	−0.03 (−0.19, 0.13)	0.7	1
Healthy plant foods	1.72 (−4.88, 8.31)	0.04 (−0.12, 0.2)	0.61	1
**Exploratory attitude outcomes**
Importance of health	0.10 (−0.10, 0.30)	0.08 (−0.08, 0.24)	0.34	1
Importance of environment	0.06 (−0.16, 0.29)	0.05 (−0.12, 0.21)	0.57	1
Importance of animal welfare	0.18 (−0.04, 0.39)	0.13 (−0.03, 0.29)	0.12	1
Interest in activism		0.17 (0.01, 0.33)	0.04	0.64
Speciesism		−0.08 (−0.24, 0.09)	0.36	1
Social dominance orientation		−0.03 (−0.2, 0.13)	0.68	1

**Table 3:** For Study 1, estimated moderation by baseline demographic variables of the intervention’s effect on the primary outcome (total MAP consumption). Raw mean differences represent ounces consumed over the past week. Main effects represent differences in average consumption by the demographic variables. Effect modification estimates represent differences in intervention effectiveness for each demographic variable, with negative values representing greater effectiveness (i.e., greater reductions in consumption). Brackets are 95% confidence intervals that do not correct for multiple testing. “Politically neutral”: Independent or “Other/I don’t know”. “County liberalism” represents a 10-percentage point higher proportion of votes cast for Democratic presidential candidates in the participant’s county.

Coefficient	Raw Mean Difference	Standardized Mean Difference	*p*-Value	Bonferroni *p*-Value
**Main effects**
Intercept	51.2 (28.99, 73.41)	1.42 (0.8, 2.04)	<0.0001	
Intervention (vs. control)	16.96 (−12.2, 46.13)	0.47 (−0.34, 1.28)	0.25	
Female	−9 (−19.08, 1.08)	−0.25 (−0.53, 0.03)	0.08	
Age years ≤25	1.71 (−7.13, 10.55)	0.05 (−0.2, 0.29)	0.7
At least 2-year college	2.66 (−5.97, 11.28)	0.07 (−0.17, 0.31)	0.54	
Caucasian	5.66 (−2.67, 13.98)	0.16 (−0.07, 0.39)	0.18	
Democrat (vs. Independent/other)	−0.21 (−14.53, 14.1)	−0.01 (−0.4, 0.39)	0.98	
Republication (vs. Independent/other)	−3.63 (−18.24, 10.98)	−0.1 (−0.51, 0.3)	0.63	
County liberalism	0.9 (−2.83, 4.62)	0.02 (−0.08, 0.13)	0.64	
**Moderation of intervention effect**
Female	−2.3 (−15.21, 10.61)	−0.06 (−0.42, 0.29)	0.73	1
Age years ≤25	−0.37 (−12.97, 12.22)	−0.01 (−0.36, 0.34)	0.95	1
At least 2-year college	−2.22 (−14.44, 9.99)	−0.06 (−0.4, 0.28)	0.72	1
Caucasian	−0.71 (−12.43, 11.01)	−0.02 (−0.34, 0.31)	0.9	1
Independent/other (vs. Republican)	−0.58 (−19.07, 17.9)	−0.02 (−0.53, 0.5)	0.95	1
Democrat (vs. Republican)	−0.75 (−19.04, 17.55)	−0.02 (−0.53, 0.49)	0.94	1
County liberalism	−2.34 (−6.71, 2.03)	−0.07 (−0.19, 0.06)	0.29	1

**Table 4:** For Study 3, demographic characteristics of the 665 participants at baseline. Continuous variables are reported as medians with 25th and 75th percentiles. Binary variables are reported as counts and percentages. Participants could indicate multiple races. “County liberalism”: in the participant’s county, the proportion of votes from the 2000–2016 United States presidential elections that went to the Democratic candidate.

Characteristic	Intervention (*n* = 333)	Control (*n* = 332)
**Sex**
Male	82 (25%)	98 (30%)
Female	251 (75%)	234 (70%)
Other	0 (0%)	0 (0%)
**Age (years)**	60 (48, 67)	58 (49, 67)
**Education**
Did not graduate high school	2 (1%)	2 (1%)
Graduated high school	28 (8%)	24 (7%)
Graduated 2-year college	32 (10%)	26 (8%)
Graduated 4-year college	146 (44%)	127 (38%)
Completed post-graduate degree	125 (38%)	153 (46%)
**Political party**
Democrat	209 (63%)	192 (58%)
Republican	15 (5%)	12 (4%)
Independent	61 (18%)	83 (25%)
Other/I don’t know	48 (14%)	45 (14%)
**County liberalism**	0.70 (0.70, 0.74)	0.70 (0.70, 0.74)
**Race**
Caucasian	258 (77%)	240 (72%)
Black/African American	6 (2%)	8 (2%)
Hispanic	26 (8%)	38 (11%)
East Asian	23 (7%)	31 (9%)
Southeast Asian	18 (5%)	18 (5%)
South Asian	12 (4%)	13 (4%)
Native American	3 (1%)	11 (3%)
Middle Eastern	2 (1%)	11 (3%)
Pacific Islander	5 (2%)	6 (2%)

**Table 5:** For Study 2, estimated intervention effects for the primary outcomes, secondary food outcomes, and exploratory attitude outcomes. Negative estimates represent intervention effects in the desired direction (reduced consumption). “Target demographic”: Participants who reported being Democrats and having graduated 2-year college. Raw mean differences represent ounces consumed over the past week for the primary outcome and secondary food outcomes; they represent units on a 7-point Likert scale for the perceived importance items; and they are omitted for the three composite scales, which were already standardized. Brackets are 95% confidence intervals without correction for multiple testing.

Outcome	Raw Mean Difference	Standardized Mean Difference	*p*-Value	Bonferroni *p*-Value
**Primary outcome**
Total meat and animal products	−2.46 (−8.78, 3.85)	−0.09 (−0.32, 0.14)	0.43	
Total meat and animal products
(target demographic)	−1.72 (−8.84, 5.41)	−0.07 (−0.34, 0.21)	0.63	
**Secondary food outcomes**
Meat	−0.97 (−4.43, 2.49)	−0.07 (−0.30, 0.16)	0.57	1
Non-meat animal products	−1.49 (−6.09, 3.12)	−0.07 (−0.28, 0.14)	0.52	1
Chicken	−0.41 (−2.49, 1.67)	−0.04 (−0.27, 0.18)	0.69	1
Turkey	0.02 (−0.5, 0.54)	0.01 (−0.2, 0.21)	0.94	1
Fish	0.05 (−1.05, 1.15)	0.01 (−0.18, 0.20)	0.93	1
Pork	−0.28 (−0.87, 0.3)	−0.08 (−0.26, 0.09)	0.34	1
Beef	−0.11 (−1.05, 0.83)	−0.02 (−0.23, 0.18)	0.81	1
Other meat	−0.25 (−0.62, 0.12)	−0.12 (−0.29, 0.06)	0.19	1
Dairy	−1.24 (−5.29, 2.8)	−0.06 (−0.26, 0.14)	0.54	1
Eggs	−0.23 (−1.76, 1.29)	−0.03 (−0.25, 0.18)	0.76	1
Healthy plant foods	5.23 (−8.3, 18.76)	0.09 (−0.14, 0.32)	0.44	1
**Exploratory attitude outcomes**
Importance of health	0.00 (−0.23, 0.23)	0.00 (−0.22, 0.21)	0.99	1
Importance of environment	0.00 (−0.26, 0.27)	0.00 (−0.20, 0.20)	0.97	1
Importance of animal welfare	0.13 (−0.26, 0.52)	0.10 (−0.20, 0.39)	0.49	1
Interest in activism		−0.05 (−0.4, 0.31)	0.78	1
Speciesism		0.08 (−0.26, 0.42)	0.62	1
Social dominance orientation		0.02 (−0.28, 0.32)	0.79	1

**Table 6:** For Study 3, the percent of intervention-group participants (*n* = 333) who pledged to reduce consumption, who pledged to eliminate consumption, and who made either pledge for each food type.

Food	“Reduce” Pledge (%)	“Eliminate” Pledge (%)	Either Pledge (%)
Chicken	40	14	53
Fish	39	7	46
Pork	31	28	58
Beef	35	23	57
Other meat	36	25	60
Dairy	36	8	44
Eggs	39	6	45

## Data Availability

All methods and statistical analyses were preregistered in detail (Study 1: https://osf.io/m3d2y/, Study 2: https://osf.io/etpvf/, Study 3: https://osf.io/n52yd/). The minor deviations from those protocols (i.e., analyses that we omitted given the null results) are disclosed in the main text or Supplement. All measures and experiments are reported, and we determined sample sizes in advance. All data (except participants’ counties of residence per our IRB approval), the documentary intervention, questionnaire materials, and analysis code are publicly available and documented (https://osf.io/8dzng/). The documentary intervention is freely available under copyright by The Humane League. Others wishing to reuse the intervention for research or educational purposes are encouraged to secure permission from The Humane League, though such uses are typically covered under fair use in the United States.

## Supplementary Materials

The following is available online at https://www.mdpi.com/2072-6643/13/12/4555/s1, File S1: Supplementary Methods and Results.
