# Correction: Mathur et al. Effectiveness of a Theory-Informed Documentary to Reduce Consumption of Meat and Animal Products: Three Randomized Controlled Experiments. *Nutrients* 2021, *13*, 4555

**DOI:** 10.3390/nu14132672

**Published:** 2022-06-28

**Authors:** Maya B. Mathur, Jacob R. Peacock, Thomas N. Robinson, Christopher D. Gardner

**Affiliations:** 1 Quantitative Sciences Unit and Department of Pediatrics, Stanford University, Stanford, CA 94305, USA; 2 The Humane League Labs, Rockville, MD 20849, USA; jacob@rethinkpriorities.org; 3 Stanford Solutions Science Lab, Department of Pediatrics, Stanford University, Stanford, CA 94305, USA; tom.robinson@stanford.edu; 4 Stanford Prevention Research Center, Stanford University, Stanford, CA 94305, USA; cgardner@stanford.edu

The authors wish to make the following correction to this paper [1]. During typesetting, the authors noted that in Table 4, the political party affiliations were listed in the wrong order, such that the counts and percentages referred to the wrong party affiliations. Table 4 should have appeared as follows:
**Characteristic****Intervention (*n* = 333)****Control (*n* = 332)****Sex**

 Male82 (25%)98 (30%) Female251 (75%)234 (70%) Other0 (0%)0 (0%)**Age (years)**60 (48, 67)58 (49, 67)**Education**

 Did not graduate high school2 (1%)2 (1%) Graduated high school28 (8%)24 (7%) Graduated 2-year college32 (10%)26 (8%) Graduated 4-year college146 (44%)127 (38%) Completed post-graduate degree125 (38%)153 (46%)**Political party**

 Democrat209 (63%)192 (58%) Republican15 (5%)12 (4%) Independent61 (18%)83 (25%) Other/I don’t know48 (14%)45 (14%)**County liberalism**0.70 (0.70, 0.74)0.70 (0.70, 0.74)**Race**

 Caucasian258 (77%)240 (72%) Black/African American6 (2%)8 (2%) Hispanic26 (8%)38 (11%) East Asian23 (7%)31 (9%) Southeast Asian18 (5%)18 (5%) South Asian12 (4%)13 (4%) Native American3 (1%)11 (3%) Middle Eastern2 (1%)11 (3%) Pacific Islander5 (2%)6 (2%)

The authors apologize for any inconvenience caused and state that the scientific conclusions are unaffected. The original publication has also been updated.
