# Supplementary material for: Effectiveness of a Theory-Informed Documentary to Reduce Consumption of Meat and Animal Products: Three Randomized Controlled Experiments
Source: Nutrients. 2021 Dec 20;13(12):4555. doi: 10.3390/nu13124555 (PMC8708224; doi:10.3390/nu13124555)
Supplement: Supplementary file 1 [file nutrients-13-04555-s001.zip › nutrients-1481460-supplementary.pdf]

Supplement: Effectiveness of a theory-informed documentary to reduce consumption of meat and animal products

Contents

1 Study 1 **3**

1.1 Sample size determination . . . 3

1.2 Duration of follow-up . . . 3

1.3 T_0 questionnaire items . . . 3

1.4 Demographic measures . . . 3

1.4.1 Attention check . . . 5

1.5 T_1 questionnaire items . . . 5

1.5.1 Food consumption . . . 5

1.5.2 Awareness probe . . . 6

1.5.3 COVID pandemic effects on dietary agency . . . 8

1.5.4 Exploratory attitude outcomes . . . 8

1.6 Details of primary statistical analyses . . . 11

1.7 Additional results of sensitivity analyses . . . 12

2 Study 2 **13**

2.1 Additional results . . . 13

3 Study 3 **15**

3.1 Sample size determination . . . 15

3.2 Additional information on recruitment 15

3.3 Choice of moderators defining the target demographic 16

3.4 T_0 intervention engagement items 16

3.5 Interrim engagement email 19

3.6 Additional results of sensitivity analyses 20

1. Study 1

1.1. Sample size determination

We chose the sample size by assuming 85% retention such that we would analyze approximately 552 participants, yielding 80% power to detect an effect size of $d = 0.24$ on the standardized mean difference scale, 95% power to detect an effect size of $d = 0.31$, and an estimated confidence interval width of $d = 0.33$. Secondarily, this sample size yields approximately 80% power to detect an interaction of the intervention with a balanced binary covariate of $d = 0.34$.

1.2. Duration of follow-up

Individual follow-up times from T_0 to T_1 varied from 12 days to 19 days because participants did not necessarily have to complete the T_1 questionnaire the day we made it available. To achieve a typical follow-up time of approximately 2 weeks, we made the follow-up questionnaire available on Prolific 12 days after the date on which we closed baseline data collection, and it remained open for 7 days.

1.3. T_0 questionnaire items

The questionnaire items are listed by chronological appearance in the questionnaire.

1.4. Demographic measures

Your sex:

- ◯ Female
- ◯ Male
- ◯ Other

Your age (years): ________

Your highest educational level:

◯ Did not graduate high school

◯ Graduated high school

◯ Graduated 2-year college

◯ Graduated 4-year college

◯ Obtained post-graduate degree (e.g., MS, MD, MBA, PhD, etc.)

Your ethnicity (check all that apply):

☐ Caucasian

☐ Hispanic

☐ Black/African American

☐ Middle Eastern

☐ Pacific Islander

☐ Native American

☐ South Asian

☐ East Asian

☐ Southeast Asian

Your current location of residence:
[drop-down menus with states and counties]

Your political party affiliation:

◯ Democrat

◯ Republican

◯ Independent

◯ Other/I don't know

For each participant, we used state and county data, along with an external database (MIT Election Data and Science Lab, 2018), to calculate "county liberalism", defined as

the proportion of votes in all 2000–2016 United States presidential elections that went to the Democratic candidate among all votes in the county that were cast for a major-party candidate.

1.4.1 Attention check

Which of the following points did the video make? Please select all that apply.

- ☐ Regularly eating seafood is good for you.
- ☐ Most Americans get less than the recommended amount of exercise.
- ☐ The ways we raise animals for human consumption causes the animals to suffer.
- ☐ It is important to teach children good habits in terms of exercise.
- ☐ Exercising for even 10 minutes a day can improve health.

1.5. T_1 questionnaire items

1.5.1 Food consumption

As follows, we modified the freely available National Cancer Institute Dietary Health Questionnaire III, specifically the 1-month version with portion sizes (*Diet History Questionnaire III (DHQ III)*, 2018; Thompson et al., 2002). Our modifications were: (1) adapting the frequency options for our briefer follow-up time frame; (2) condensing individual foods into fewer categories, informed by those used in Macdonald et al. (2016); and (3) omitting a large number of foods not relevant to the present research.

We examined 15 total foods, presented in this order:

- Healthy plant-based foods (5): leafy green vegetables, other vegetables, fruits, whole grains, legumes (including beans)
- Meats (6): chicken, turkey, fish, pork, beef, other meat
- Other animal products (2): dairy, eggs
- Decoy foods (nutritionally undesirable plant-based foods) (2): refined grains (e.g., white bread), sweetened beverages such as soft drinks

For each food, we asked the following questions:

Over the past week, how often did you eat **[FOOD]**?

- ◯ Never
- ◯ 1 time in the past week
- ◯ 2 times in the past week
- ◯ 3-4 times in the past week
- ◯ 5-6 times in the past week
- ◯ 1 time per day
- ◯ 2 or more times per day

Each time you ate **[FOOD]**, how much did you usually eat?

- ◯ Less than 2 ounces or less than 1/2 cup
- ◯ 2 to 5 ounces or 1/2 to 1 cup
- ◯ More than 5 ounces or more than 1 cup

For analysis purposes, we expressed consumption amounts in terms of ounces per week using midpoints of each category (e.g., 3.5 for “3-4 times in the past week”), with the exception of the upper categories for frequency and amount. For these, “2 or more times per day” was coded as 2, and “more than 5 ounces or 1 cup” was coded as 5.

1.5.2 Awareness probe

We assessed participants’ potential awareness of the study’s purpose using 2 multiple-choice items that mimicked a funnel debriefing. The first item asked, “What do you think the researchers doing this study were most interested in measuring?”, with 10 possible responses consisting of different macronutrients and food categories. The second item asked participants why they thought the researchers measured the chosen item: to look at general consumption patterns, to increase consumption of this item, or to decrease consumption. We coded those participants who responded correctly to both items (i.e., “meat and animal product intake” followed by “to try to decrease people’s meat and animal product intake”) as “potentially aware”.

What do you think the researchers doing this study were most interested in measuring?

- ◯ Total caloric intake
- ◯ Protein intake
- ◯ Fat intake
- ◯ Vegetable intake
- ◯ Fruit intake
- ◯ Whole-grain intake
- ◯ Meat and animal product intake
- ◯ Refined-grain intake
- ◯ Sweetened beverage intake
- ◯ I don't know what the researchers were interested in measuring

For participants who did not answer "I don't know", the below questions were presented with the participant's chosen response above replacing "[CHOSEN ITEM]":

Why do you think this study measured **[CHOSEN ITEM]** intake?

- ◯ To look at general consumption patterns and see how they vary across different demographic groups
- ◯ To try to increase people's **[CHOSEN ITEM]** intake
- ◯ To try to decrease people's **[CHOSEN ITEM]** intake
- ◯ I don't know why the study measured this

1.5.3 COVID pandemic effects on dietary agency

In the past week, how much has the coronavirus pandemic affected your ability to choose what you eat?

1	2	3	4	5	6	7
Much less choice	Somewhat less choice	Slightly less choice	No change	Slightly more choice	Somewhat more choice	Much more choice

1.5.4 Exploratory attitude outcomes

We collected exploratory measures of participants' attitudes and values regarding the 3 harms of animal product consumption highlighted in the documentary (health, the environment, and animal welfare) by asking participants to rate on a 7-point scale how important they perceived each issue to be (Hekler et al., 2010). We also probed in more detail participants' attitudes regarding animal welfare because this type of educational appeal has appeared only relatively recently in dietary interventions (Mathur et al., 2021) and has received less academic study than appeals regarding health or the environment (Bianchi et al., 2018; Grundy et al., 2021). To this end, we assessed participants' interest in animal welfare activism and reform (Corning & Myers, 2002), and we collected a measure of "speciesism", which is the assignment of a different moral standing based on species membership itself, holding constant ethically relevant traits such as sentience and the capacity to suffer (Caviola et al., 2019; Singer, 1995). Finally, we assessed participants' social dominance orientation, which is a generalized preference for group-based hierarchy and inequality that strongly predicts prejudice against a number of human social groups (e.g., poor people and ethnic minorities) and of support for policies that uphold social hierarchies (Ho et al., 2015). We collected this measure because both speciesism (Caviola et al., 2019) and meat-eating habits (Allen et al., 2000) are cross-sectionally associated with social dominance orientation, leading us to speculate that intervening on attitudes toward animals might reduce social dominance orientation. The attitude measures appeared after the food consumption items and awareness probes because we expected that the attitude measures themselves could increase participants' awareness of the purpose of the study. The activism, speciesism, and social dominance scales used in analysis were standardized sums of multiple items.

Perceived importance of health, the environment, and animal welfare We used items minimally adapted from Hekler et al. (2010). We treated these three items as separate outcomes.

For each of the issues below, think about how important the issue is to you, compared to other things in your life. Show how important the issue is to you on the scale below.

1	2	3	4	5	6
Not at all important compared to other things in your life	Less important	About as important	More important	Just about the most important	The very most important

1. Eating a healthful diet
2. Environmental sustainability
3. Animal welfare

Speciesism We used without modification the scale developed and validated by Caviola et al. (2019). Participants rated each item on a 7-point scale from "strongly disagree" to "strongly agree".

1. Morally, animals always count for less than humans.
2. Humans have the right to use animals however they want to.
3. It is morally acceptable to keep animals in circuses for human entertainment.
4. It is morally acceptable to trade animals like possessions.
5. Chimpanzees should have basic legal rights such as a right to life or a prohibition of torture. (reverse-coded)
6. It is morally acceptable to perform medical experiments on animals that we would not perform on any human.

Interest in animal welfare activism and reform The first two items are modified from those with the largest eigenvalues from the 35-item Activism Orientation Scale (Corning & Myers, 2002); we modified them to pertain specifically to animal advocacy. We created the remaining three items to relate to concrete actions relevant to animal advocacy. participants rated each item on a 7-point Likert scale ranging from "extremely unlikely" to "extremely likely" (Corning & Myers, 2002).

How likely is it that you will engage in this activity in the future?

1. Display a poster or bumper sticker with a message about animal welfare issues, such as factory farming?
2. Invite a friend to attend a meeting of an organization or event focused on animal welfare issues, such as factory farming?
3. Vote in favor of legislation that would improve animal welfare conditions on factory farms?
4. Sign a petition to a major grocery store asking them to stop selling products produced in factory farms?
5. Donate to an organization or event focused on animal welfare issues, such as factory farming?

Social dominance orientation We used without modification Ho et al. (2015)'s validated 8-item $SDO_{7(s)}$ scale.

Show how much you favor or oppose each idea below by selecting a number from 1 to 7 on the scale below. You can work quickly; your first feeling is generally best.

1	2	3	4	5	6	7
Strongly oppose	Somewhat oppose	Slightly oppose	Neutral	Slightly favor	Somewhat favor	Strongly favor

1. An ideal society requires some groups to be on top and others to be on the bottom.
2. Some groups of people are simply inferior to other groups.
3. No one group should dominate in society.
4. Groups at the bottom are just as deserving as groups at the top.
5. Group equality should not be our primary goal.
6. It is unjust to try to make groups equal.
7. We should do what we can to equalize conditions for different groups.
8. We should work to give all groups an equal chance to succeed.

1.6. Details of primary statistical analyses

Multiple imputation We used fully conditional multiple imputation for all variables with missing data, including the outcome, using the R package `mice` (Buuren & Groothuis-Oudshoorn, 2010; Van Buuren, 2018). We created 10 imputations (Graham et al., 2007), pooled variances across imputations using Rubin's Rules, and conducted hypothesis testing on the pooled estimates using the t-distribution (Dong & Peng (2013)'s Equations (8-9)).

Calculation of standardized mean differences We calculated standardized mean differences using the Hedges' g metric (Hedges, 1981), which represent intervention effects in terms of the conditional standard deviation of the outcome, $\sigma_{Y \mid X}$, where Y is the outcome and X is the intervention indicator. When calculating standardized mean differences for moderators, we again simply standardized by $\sigma_{Y \mid X}$. We could have standardized by the estimated residual standard deviation in the multivariable regression model, which would additionally have conditioned on the moderators and hence yielded larger standardized mean differences. We used $\sigma_{Y \mid X}$ for comparability with the standardized mean differences of the primary analyses.

Analysis of attention check item Instrumental variables analysis makes the excludability assumption Angrist et al. (1996), namely that if the intervention affected an individual participant's consumption, it did so by first causing the participant to become aware of the documentary's content, as measured by the attention check item. This assumption entails, for example, that the documentary did not have any entirely subconscious means of affecting consumption that in no way required participants to be aware of the documentary's contents.

We did not conduct analyses that controlled for passing the attention check, nor did we conduct subset analyses using attentive participants. Both types of analyses are highly problematic; by controlling for a post-intervention variable, they do not estimate any interpretable treatment effect (Aronow et al., 2019). For example, such analyses do *not* estimate the treatment effect among attentive participants.

1.7. Additional results of sensitivity analyses

Table S1 shows complete-case estimates of the intervention's effects on the primary outcome and all additional outcomes.

Outcome	Raw diff.	SMD	p-value
**Primary outcome**			
Total meat and animal products	-0.37 [-6.38, 5.64]	-0.01 [-0.16, 0.14]	0.90
**Secondary food outcomes**			
Meat consumption	-1.03 [-5.34, 3.27]	-0.04 [-0.19, 0.11]	0.64
Non-meat animal products	0.66 [-2.54, 3.86]	0.03 [-0.12, 0.19]	0.68
Chicken	0.08 [-1.68, 1.84]	0.01 [-0.15, 0.16]	0.93
Turkey	-0.50 [-1.41, 0.42]	-0.09 [-0.24, 0.06]	0.29
Fish	0.10 [-0.98, 1.18]	0.02 [-0.14, 0.17]	0.86
Pork	-0.13 [-1.04, 0.79]	-0.02 [-0.18, 0.13]	0.79
Beef	-0.46 [-1.72, 0.80]	-0.06 [-0.21, 0.09]	0.48
Other meat	-0.13 [-0.97, 0.72]	-0.02 [-0.18, 0.13]	0.77
Dairy	0.99 [-1.66, 3.63]	0.06 [-0.09, 0.22]	0.46
Eggs	-0.33 [-1.76, 1.11]	-0.04 [-0.19, 0.12]	0.66
Healthy plant foods	2.05 [-4.6, 8.71]	0.05 [-0.1, 0.2]	0.54
**Exploratory attitude outcomes**			
Importance of health	0.09 [-0.10, 0.29]	0.08 [-0.07, 0.23]	0.34
Importance of environment	0.07 [-0.14, 0.28]	0.06 [-0.10, 0.21]	0.49
Importance of animal welfare	0.19 [-0.02, 0.40]	0.15 [0, 0.31]	0.07
Interest in activism	–	0.19 [0.04, 0.34]	0.02
Speciesism	–	-0.08 [-0.23, 0.07]	0.34
Social dominance orientation	–	-0.03 [-0.18, 0.12]	0.72

Table S1: *For Study 1, complete-case estimates of intervention effects for the primary outcome, secondary food outcomes, and exploratory attitude outcomes. Negative estimates represent intervention effects in the desired direction (reduced consumption). Raw mean differences ("raw diff.") represent ounces consumed over the past week for the primary outcome and secondary food outcomes; they represent units on a 7-point Likert scale for the perceived importance items; and they are omitted for the three composite scales, which were already standardized. SMD: standardized mean difference. Brackets are 95% confidence intervals without correcting for multiple testing. p-values are not corrected for multiple testing.*

2. Study 2

2.1. Additional results

Table S2 shows participant demographics, Table S3 shows moderation estimates, and Table S4 shows estimated intervention effects on past-week food outcomes.

Characteristic	Intervention (n=148)	Control (n=152)
**Sex**		
Male	80 (54%)	83 (55%)
Female	67 (45%)	68 (45%)
Other	1 (1%)	1 (1%)
**Age (years)**	33 (26, 41)	32 (27, 42)
**Education**		
Did not graduate high school	1 (1%)	2 (1%)
Graduated high school	34 (23%)	42 (28%)
Graduated 2-year college	23 (16%)	13 (9%)
Graduated 4-year college	56 (38%)	55 (36%)
Completed post-graduate degree	34 (23%)	40 (26%)
**Political party**		
Democrat	83 (56%)	86 (57%)
Independent	28 (19%)	30 (20%)
Republican	28 (19%)	29 (19%)
Other/I don't know	9 (6%)	7 (5%)
**County liberalism**	0.57 (0.44, 0.70)	0.55 (0.45, 0.70)
**Race**		
Causasian	108 (73%)	115 (76%)
Black/African American	14 (9%)	24 (16%)
Hispanic	11 (7%)	6 (4%)
East Asian	9 (6%)	5 (3%)
Southeast Asian	6 (4%)	5 (3%)
South Asian	3 (2%)	3 (2%)
Native American	5 (3%)	3 (2%)
Middle Eastern	3 (2%)	2 (1%)

Table S2: *For Study 2, demographic characteristics of the 300 participants at baseline. Continuous variables are reported as medians with 25th and 75th percentiles. Binary variables are reported as counts and percentages. Participants could indicate multiple races. "County liberalism": in the participant's county, the proportion of votes from the 2000–2016 United States presidential elections that went to the Democratic candidate.*

Coefficient	Raw diff.	SMD	p-value
**Main effects**			
Intercept	0.45 [-0.16, 1.06]	0.38 [-0.14, 0.9]	0.15
Intervention (vs. control)	0.42 [-0.98, 1.83]	0.36 [-0.83, 1.55]	0.55
Female	0.18 [-0.08, 0.44]	0.15 [-0.07, 0.37]	0.17
Age $\leq$ 25 years	0.06 [-0.26, 0.38]	0.05 [-0.22, 0.32]	0.70
At least 2-year college	0.11 [-0.17, 0.38]	0.09 [-0.14, 0.33]	0.45
Caucasian	-0.10 [-0.45, 0.25]	-0.09 [-0.39, 0.21]	0.58
Independent/other (vs. Republican)	-0.12 [-0.52, 0.28]	-0.10 [-0.44, 0.24]	0.56
Democrat (vs. Republican)	-0.29 [-0.63, 0.06]	-0.24 [-0.54, 0.05]	0.10
County liberalism	-0.02 [-0.11, 0.06]	-0.02 [-0.09, 0.06]	0.63
**Moderation of intervention**			
Female	0.08 [-0.44, 0.60]	0.07 [-0.37, 0.51]	0.76
Age $\leq$ 25 years	-0.08 [-0.63, 0.48]	-0.06 [-0.54, 0.41]	0.79
At least 2-year college	-0.07 [-0.65, 0.51]	-0.06 [-0.55, 0.43]	0.81
Caucasian	-0.07 [-0.64, 0.5]	-0.06 [-0.55, 0.42]	0.80
Independent/other (vs. Republican)	0.16 [-0.57, 0.88]	0.13 [-0.48, 0.75]	0.67
Democrat (vs. Republican)	0.17 [-0.53, 0.86]	0.14 [-0.45, 0.73]	0.64
County liberalism	0.05 [-0.11, 0.21]	0.04 [-0.1, 0.18]	0.56

Table S3: *In Study 2, estimated moderation by baseline demographic variables of intervention effects on the primary outcome (the continuous measure of intentions to reduce consumption). Positive estimates represent intervention effects in the desired direction (increased intentions to reduce consumption). Raw mean differences represent ounces consumed over the past week. Main effects represent differences in average consumption by the demographic variables. SMD: Standardized mean difference (Hedges' g). Moderation estimates represent differences in intervention effectiveness for each demographic variable, with negative values representing greater effectiveness (i.e., greater reductions in consumption). Brackets are 95% confidence intervals that do not correct for multiple testing. "County liberalism" represents a 10-percentage point higher proportion of votes cast for Democratic presidential candidates in the participant's county.*

Outcome	Raw diff.	SMD	p-value
**Past food outcomes**			
Total meat and animal products	-4.07 [-11.74, 3.60]	-0.12 [-0.35, 0.11]	0.30
Meat consumption	-3.57 [-8.76, 1.63]	-0.15 [-0.38, 0.07]	0.18
Non-meat animal products	-0.50 [-4.94, 3.93]	-0.03 [-0.25, 0.20]	0.82
Chicken	-2.14 [-3.93, -0.35]	-0.27 [-0.50, -0.04]	0.02
Turkey	-1.08 [-2.23, 0.07]	-0.21 [-0.44, 0.02]	0.07
Fish	-0.12 [-2.19, 1.96]	-0.01 [-0.24, 0.21]	0.91
Pork	0.41 [-0.56, 1.39]	0.10 [-0.13, 0.32]	0.41
Beef	-0.47 [-1.89, 0.95]	-0.08 [-0.30, 0.15]	0.52
Other meat	-0.17 [-1.18, 0.84]	-0.04 [-0.27, 0.19]	0.74
Dairy	0.77 [-2.85, 4.38]	0.05 [-0.18, 0.28]	0.68
Eggs	-1.27 [-3.47, 0.93]	-0.13 [-0.36, 0.10]	0.26
Healthy plant foods	10.87 [-0.41, 22.15]	0.22 [-0.01, 0.45]	0.06
**Exploratory attitude outcomes**			
Importance of health	-0.01 [-0.28, 0.26]	-0.01 [-0.23, 0.22]	0.96
Importance of environment	0.30 [-0.01, 0.61]	0.22 [-0.01, 0.45]	0.05
Importance of animal welfare	0.22 [-0.07, 0.51]	0.17 [-0.05, 0.40]	0.13
Interest in activism	–	0.16 [-0.07, 0.39]	0.17
Speciesism	–	-0.05 [-0.28, 0.18]	0.66
Social dominance orientation	–	-0.02 [-0.25, 0.21]	0.86

Table S4: *In Study 2, estimated intervention effects on secondary outcomes. Raw mean differences represent ounces consumed over the past week for the primary outcome and secondary food outcomes; they represent units on a 7-point Likert scale for the perceived importance items; and they are omitted for the three composite scales, which were already standardized. SMD: Standardized mean difference (Hedges' g). Brackets are 95% confidence intervals that do not correct for multiple testing.*

3. Study 3

3.1. Sample size determination

We chose the sample size a priori to yield 80% power to detect an effect size of $SMD = 0.32$ and 95% power to detect an effect size of $SMD = 0.42$.

3.2. Additional information on recruitment

The registry from which we recruited participants was built over 20 years and approximately 20 human nutrition intervention trials run by the Gardner Nutrition Studies Research Group

at Stanford University. The registry includes participants who at at some point had completed an online screener to be a potential participant in at least one of the past trials, and agreed to allow their contact information to be kept for the purpose of being informed about future studies. Approximately 25% of individuals in the registry are past study participants. The remaining individuals either had not been eligible for the particular study for which they were screened, or who had been eligible but ultimately chose not to participate after learning about the details of participation. With every new announcement to the registry of new research participation opportunities, those receiving the notification are informed that they can be removed from the registry simply be replying to the notification with this request.

We sent invitation emails at T_0 in batches over several weeks due to restrictions on the number of emails we could send at once, but we ensured that each participant in the T_0 questionnaire received the invitation to participate in T_1 14 days after the date they completed T_0.

3.3. Choice of moderators defining the target demographic

We defined the target demographic as having completed at least 2-year college and being a Democrat because, in our initial analyses of Study 1, the moderation estimates were strongest for these 2 variables. After collecting data for all 3 studies, we finalized all the analysis code. During this process, we made modifications to the multiple imputation model to resolve warnings we received from a newly updated version of the imputation software package. (Specifically, we had originally performed imputation on the raw data before creating derived variables such as total consumption, whereas in the final analyses, we calculated the derived variables first before performing imputation.) The resulting, final moderation estimates differed somewhat from those we obtained in the initial analyses. In the final analyses, having completed least 2-year college and being a Democrat were still directionally consistent with increased intervention effects but no longer had the strongest estimates of all moderators we investigated.

3.4. T_0 intervention engagement items

For participants in the intervention group only, the documentary was presented as 3 sequential chunks (corresponding to content about health, the environment, and animal welfare), each followed by questions about its content as follows.

After the health component of the documentary:

> As discussed in the video, plant-based diets can have several health benefits. Can you name at least 2 of these benefits that you found compelling?
>
> *[Free-text box]*

After the environment component of the documentary:

> As discussed in the video, the production of meat and animal products causes several types of environmental damage. Can you name at least 2 of these damages that you found compelling?
>
> *[Free-text box]*

After the animal welfare component of the documentary:

> As discussed in the video, the production of meat and animal products causes significant suffering to animals in many ways. Can you name at least 2 ways in which farmed animals suffer that you found compelling?
>
> *[Free-text box]*

For participants assigned to view the documentary, we asked following questions after participants viewed the documentary.

> Many of our previous Stanford research participants have pledged to eat and drink less meat and animal products after watching this documentary. Are you willing to take one of the following pledges?
>
> *[Matrix of radio buttons with foods listed as rows (chicken, fish, pork, beef, all other meats, eggs, dairy) and pledges listed as columns ("Yes, I pledge to stop eating this food", "Yes, I pledge to eat this food less often", "No, I do not wish to make a pledge about this food")]*

Participants who chose one of the pledges then completed the following items.

> Congratulations on making your pledge.
>
> Research shows that making concrete goals helps us to follow through on our intentions to change our behavior. A good goal is specific, measurable, and attainable, and has a deadline.
>
> For example, "I will stop eating all meat, eggs, and dairy by XXX date". Please pick a date on which you plan to meet your pledge goal.
>
> I will meet my pledge goal by the following date: *[Clickable calendar]*

Research also shows that how we eat is largely habitual. It can be initially hard to break out of old habits, but once we form new habits, they can become just as automatic and easy to maintain as the old habits they replace.

What specific strategies will you try in order to form a habit around your new goals? Please check as many as you would like. These are just examples – use your imagination!

- ☐ On the same day every week, I will prepare several servings of plant-based meals so that I don't have to think about it during the weekday.
- ☐ For a specific meal (e.g., weekday lunches), I will always buy take-out from restaurants that have plant-based options.
- ☐ I will meet my pledge goal a gradual way by eliminating one meat or animal product at a time (e.g., first chicken, then eggs, etc.).
- ☐ I will choose a specific day of the week (e.g., Monday) when I will eat only plant-based meals.
- ☐ I will choose a specific meal (e.g., dinner) when I will eat only plant-based meals.
- ☐ Other (please specify): ______________________________

Last, one of the most effective ways to form new dietary habits is to keep a diary to track what you are eating. Our participants tell us this has been a very helpful way to meet their pledge goals.

For example, you could have a calendar and check off each day you were able to meet your pledge goal – try to make the longest streak you can! There are also smartphone apps for this purpose (e.g., Streaks, Habit List, Chains.cc, and many more).

3.5. Interrim engagement email

One week after random assignment, we again emailed pledge-makers. The email was titled "Remember your meat pledge last week?", and its content was:

> A week ago, you watched a documentary about how meat and animal products are produced, and you joined many of our Stanford participants in making a pledge to eat less meat, eggs, and/or dairy.
>
> You pledged to stop eating the following foods: *[Piped text from matrix question]*
>
> You pledged to eat less of the following foods: *[Piped text from matrix question]*
>
> You planned to meet your pledge goals by this following date: *[Piped text from date question]*
>
> And you planned to try the following strategies to meet your pledge goals: *[Piped text from strategies question]*
>
> Keep it up! It takes about 2 weeks for new habits to start to feel easy, so you are already halfway there.

3.6. Additional results of sensitivity analyses

Table S5 shows complete-case estimates of the intervention's effects on the primary outcome and all additional outcomes.

Outcome	Raw diff.	SMD	p-value
**Primary outcomes**			
Total meat and animal products	-5.37 [-13.33, 2.59]	-0.18 [-0.44, 0.09]	0.19
Total meat and animal products (target demographic)	-3.66 [-12.89, 5.56]	-0.14 [-0.48, 0.21]	0.43
**Secondary food outcomes**			
Meat consumption	-2.34 [-6.67, 1.99]	-0.14 [-0.41, 0.12]	0.29
Non-meat animal products	-3.03 [-8.89, 2.82]	-0.14 [-0.4, 0.13]	0.31
Chicken	-1.58 [-3.71, 0.55]	-0.19 [-0.46, 0.07]	0.14
Turkey	0.04 [-0.61, 0.7]	0.02 [-0.25, 0.28]	0.89
Fish	0.74 [-0.82, 2.29]	0.13 [-0.14, 0.39]	0.35
Pork	-0.77 [-1.67, 0.14]	-0.22 [-0.49, 0.04]	0.10
Beef	-0.16 [-1.65, 1.33]	-0.03 [-0.29, 0.24]	0.84
Other meat	-0.61 [-1.28, 0.05]	-0.24 [-0.5, 0.02]	0.07
Dairy	-2.62 [-7.66, 2.42]	-0.14 [-0.4, 0.13]	0.31
Eggs	-0.41 [-2.57, 1.74]	-0.05 [-0.32, 0.21]	0.71
Healthy plant foods	7.45 [-7.54, 22.45]	0.13 [-0.13, 0.4]	0.33
**Exploratory attitude outcomes**			
Importance of health	0.00 [-0.29, 0.29]	0.00 [-0.27, 0.27]	0.99
Importance of environment	0.01 [-0.3, 0.32]	0.01 [-0.26, 0.28]	0.96
Importance of animal welfare	0.29 [-0.05, 0.64]	0.23 [-0.04, 0.5]	0.09
Interest in activism	–	0.08 [-0.19, 0.35]	0.58
Speciesism	–	0.07 [-0.2, 0.34]	0.62
Social dominance orientation	–	-0.01 [-0.29, 0.26]	0.92

Table S5: *In Study 3, complete-case estimates of intervention effects for the primary outcomes, secondary food outcomes, and exploratory attitude outcomes. Negative estimates represent intervention effects in the desired direction (reduced consumption). "Target demographic": Participants who reported being Democrats with at least a 2-year college education. Raw mean differences ("raw diff.") represent ounces consumed over the past week for the primary outcome and secondary food outcomes; they represent units on a 7-point Likert scale for the perceived importance items; and they are omitted for the three composite scales, which were already standardized. "SMD": standardized mean difference. Brackets are 95% confidence intervals that do not correct for multiple testing. p-values are not corrected for multiple testing.*

References

Allen, M. W., Wilson, M., Ng, S. H., & Dunne, M. (2000). Values and beliefs of vegetarians and omnivores. *The Journal of Social Psychology*, *140*(4), 405–422.

Angrist, J. D., Imbens, G. W., & Rubin, D. B. (1996). Identification of causal effects using instrumental variables. *Journal of the American Statistical Association*, *91*(434), 444–455.

Aronow, P. M., Baron, J., & Pinson, L. (2019). A note on dropping experimental subjects who fail a manipulation check. *Political Analysis*, *27*(4), 572–589.

Bianchi, F., Dorsel, C., Garnett, E., Aveyard, P., & Jebb, S. A. (2018). Interventions targeting conscious determinants of human behaviour to reduce the demand for meat: A systematic review with qualitative comparative analysis. *International Journal of Behavioral Nutrition and Physical Activity*, *15*(1), 102.

Buuren, S. v., & Groothuis-Oudshoorn, K. (2010). mice: Multivariate imputation by chained equations in R. *Journal of Statistical Software*, 1–68.

Caviola, L., Everett, J. A., & Faber, N. S. (2019). The moral standing of animals: Towards a psychology of speciesism. *Journal of Personality and Social Psychology*, *116*(6), 1011.

Corning, A. F., & Myers, D. J. (2002). Individual orientation toward engagement in social action. *Political Psychology*, *23*(4), 703–729.

Diet History Questionnaire III (DHQ III) (Tech. Rep.). (2018, March). National Cancer Institute. (https://epi.grants.cancer.gov/dhq3/)

Dong, Y., & Peng, C.-Y. J. (2013). Principled missing data methods for researchers. *SpringerPlus*, *2*(1), 222.

Graham, J. W., Olchowski, A. E., & Gilreath, T. D. (2007). How many imputations are really needed? some practical clarifications of multiple imputation theory. *Prevention Science*, *8*(3), 206–213.

Grundy, E., Slattery, P., Saeri, A. K., Watkins, K., Houlden, T., Farr, N., ... others (2021). Interventions that influence animal-product consumption: A meta-review. *Future Foods*. (Preprint retrieved from `https://osf.io/mcdsq/`)

Hedges, L. V. (1981). Distribution theory for Glass's estimator of effect size and related estimators. *Journal of Educational Statistics*, *6*(2), 107–128.

Hekler, E. B., Gardner, C. D., & Robinson, T. N. (2010). Effects of a college course about food and society on students' eating behaviors. *American Journal of Preventive Medicine*, *38*(5), 543–547.

Ho, A. K., Sidanius, J., Kteily, N., Sheehy-Skeffington, J., Pratto, F., Henkel, K. E., ... Stewart, A. L. (2015). The nature of social dominance orientation: Theorizing and measuring preferences for intergroup inequality using the new SDO_7 scale. *Journal of Personality and Social Psychology*, *109*(6), 1003.

Macdonald, B., Caldwell, K., & Boese, G. (2016). *The effects of 'reduce' and 'eliminate' appeals on individual meat consumption* (Tech. Rep.). Reducetarian Foundation. (https://osf.io/nxrx3/)

Mathur, M. B., Peacock, J., Reichling, D. B., Nadler, J., Bain, P. A., Gardner, C. D., & Robinson, T. N. (2021). Interventions to reduce meat consumption by appealing to animal welfare: Meta-analysis and evidence-based recommendations. *Appetite*, *164*, 105277.

MIT Election Data and Science Lab. (2018). *County Presidential Election Returns 2000-2016.* Harvard Dataverse. Retrieved from https://doi.org/10.7910/DVN/VOQCHQ doi: 10.7910/DVN/VOQCHQ

Singer, P. (1995). *Animal liberation.* Random House.

Thompson, F. E., Subar, A. F., Brown, C. C., Smith, A. F., Sharbaugh, C. O., Jobe, J. B., ... Ziegler, R. G. (2002). Cognitive research enhances accuracy of food frequency questionnaire reports: results of an experimental validation study. *Journal of the American Dietetic Association*, *102*(2), 212–225.

Van Buuren, S. (2018). *Flexible imputation of missing data.* CRC press.
